# The Early Fetal Development of Human Neocortical GABAergic Interneurons

**DOI:** 10.1093/cercor/bht254

**Published:** 2013-09-18

**Authors:** Nahidh Al-Jaberi, Susan Lindsay, Subrot Sarma, Nadhim Bayatti, Gavin J. Clowry

**Affiliations:** 1 Institute of Neuroscience; 2 Institute of Genetic Medicine, Newcastle University, Newcastle upon Tyne NE1 3BZ, UK; 3 Current address: Sheffield Institute for Translational Neuroscience (SITraN), Department of Neuroscience, University of Sheffield, Sheffield S10 2HQ, UK

**Keywords:** cerebral cortex, DLX genes, GABA, GABRB3, inhibitory interneurons, neurodevelopmental disorders

## Abstract

GABAergic interneurons are crucial to controlling the excitability and responsiveness of cortical circuitry. Their developmental origin may differ between rodents and human. We have demonstrated the expression of 12 GABAergic interneuron-associated genes in samples from human neocortex by quantitative rtPCR from 8 to 12 postconceptional weeks (PCW) and shown a significant anterior to posterior expression gradient, confirmed by in situ hybridization or immunohistochemistry for *GAD1* and *2, DLX1, 2,* and *5, ASCL1, OLIG2*, and *CALB2*. Following cortical plate (CP) formation from 8 to 9 PCW, a proportion of cells were strongly stained for all these markers in the CP and presubplate. ASCL1 and DLX2 maintained high expression in the proliferative zones and showed extensive immunofluorescent double-labeling with the cell division marker Ki-67. CALB2-positive cells increased steadily in the SVZ/VZ from 10 PCW but were not double-labeled with Ki-67. Expression of GABAergic genes was generally higher in the dorsal pallium than in the ganglionic eminences, with lower expression in the intervening ventral pallium. It is widely accepted that the cortical proliferative zones may generate CALB2-positive interneurons from mid-gestation; we now show that the anterior neocortical proliferative layers especially may be a rich source of interneurons in the early neocortex.

## Introduction

The developmental origin of GABAergic interneurons in the neocortex in primates is controversial. While it is established that, in mice, these interneurons are born almost entirely outside the neocortex (dorsal pallium) in the ganglionic eminences (GE) and associated structures such as the septum and preoptic area (subpallium) from which they migrate tangentially into the cortex (De Carlos et al. 1996; Parnavelas 2000; Marín and Rubenstein 2003; Welagen and Anderson 2011) in human, there is evidence for significant intracortical production. Immunohistochemistry (IHC) and retroviral labeling of slice preparations showed that between 10 and 25 postconceptional weeks (PCW) the majority of GABAergic interneurons are generated within the cortical progenitor zones (Letinic et al. 2002). This interpretation was supported by double-labeled immunohistochemical studies (Zecevic et al. 2005; Mo and Zecevic 2008) and in the analysis of malformations that involve deletion of the GE (Fertuzinhos et al. 2009). Furthermore, interneurons can be generated in vitro from human, but not rodent, cortical radial glial progenitor cells (Yu and Zecevic 2011). Studies in the macaque have widened these observations to other primates (Petanjek et al. 2009). However, a study of birth-dated neurons in 15 PCW human cortical cultures did not reveal GABA immunoreactive postmitotic cells in the subventricular zone (SVZ) of the cortical wall despite the identification of progenitor cells expressing the early interneuron marker ASCL1 (Hansen et al. 2010) whereas cells double-labeled for the cell division marker Ki67 and CALB2 (calretinin) were detected histologically in the 20 PCW fetus (Zecevic et al. 2011). This discrepancy may point to differences in timing and location of expression of GABA-related genes during early human fetal cortical development (Molnár and Clowry 2012).

In the rodent, parvalbumin- and somatostatin-positive GABAergic neurons arise from ventral and dorsal parts of the medial ganglionic eminence (MGE), respectively, initially entering the neocortex by tangential migration into the frontal two thirds of the neocortex, whereas Calb2-positive interneurons arise from the caudal ganglionic eminence (CGE) initially entering more caudal regions before populating the whole neocortex (Xu et al. 2004; Butt et al. 2005; Wonders and Anderson 2005; Ghanem et al. 2007; Faux et al. 2012). In the human neocortex, the numbers of CALB2-positive neurons increases from about 12 PCW, particularly, in the SVZ (Meyer et al. 2002; Bayatti, Moss et al. 2008; Zecevic et al. 2011) and interestingly, at this stage of development, the rostral neocortex is far more densely populated with CALB2 neurons than the caudal pole, suggesting that they were unlikely to arise by migration from the CGE (Bayatti, Moss et al. 2008; Zecevic et al. 2011). CALB2 interneurons are more prevalent in the adult primate than in the rodent (Conde et al. 1994; Gabbott et al. 1997; Wonders and Anderson 2005). There are other interneuron subtypes, such as the calbindin-positive double bouquet cell, which are numerous in primate, but missing or greatly reduced in other species (Ballesteros Yáñez et al. 2005). GABAergic interneurons play a crucial role in cognitive processing, fine-tuning the oscillations in neural activity in distributed networks that underlie learning and memory (Wang, Dye et al. 2010; Whittington et al. 2011). Failures in proliferation and migration of specific classes of GABAergic interneurons have been implicated in diverse conditions including autism, epilepsy, and schizophrenia (De Felipe 1999; Lewis et al. 2005; Uhlhaas and Singer 2010; Marín 2012). We must question whether mouse models are appropriate for the study of these diseases (Jones 2009; Clowry et al. 2010).

We have previously carried out a microarray study of differential gene expression between the anterior and posterior poles of the human neocortex at 8–12 PCW (Ip et al. 2010). One striking finding was that a large number of genes associated with GABAergic neurotransmission appeared to be more highly expressed anteriorly than posteriorly (see Table 1). In the present study, we have set out to better determine the relative levels of expression of a subset of these genes by carrying out quantitative real-time polymerase chain reaction (qPCR) on RNA samples collected between 8 and 12 PCW from anterior and posterior neocortex. We have compared these results with those obtained by microarray (Ip et al. 2010) and whole RNA sequencing (http://brainspan.org/rnaseq/search/index.html [date last accessed; 6 September 2013]). Then we studied expression in human tissue sections using in situ hybridization (ISH) and IHC to determine the location and type of cell expressing these genes, as well as confirming expression gradients.

**Table 1:** Regionalized expression of GABAergic genes in the human neocortex 8–12.5 PCW from U133 plus 2.0 Array Affymetrix gene chip study (Ip et al. 2010)

Fold difference A > P	Gene name	Description (from rodent studies)
7.81	DLX5	GABA interneuron progenitor: transcription factor
7.40	DLX1	GABA interneuron progenitor: transcription factor
6.57	DLX2	GABA interneuron progenitor transcription factor
6.49	ISL1	LGE-derived progenitor transcription factor
5.53	OLIG1	GABA interneuron/oligodendrocyte progenitor marker
3.71	DLX6	GABA interneuron phenotype induction transcription factor
4.77	OLIG2	GABA interneuron/oligodendrocyte progenitor marker
3.42	GAD1	GABA synthesizing enzyme (GAD 67)
3.15	NKX2.1	Subpallial GABA interneuron progenitor transcription factor
2.47	LHX6	MGE-derived GABA interneuron progenitor transcription factor
2.32	NPYR5	NPY receptor, GABA interneuron marker
2.28	ER81	Dorsal LGE interneuron progenitor transcription factor
2.09	GABRB3	GABA A receptor β subunit
1.99	VIAAT	Inhibitory amino acid transporter (Glycine/GABA)
1.88	ASCL1	GABA interneuron progenitor transcription factor
1.85	NKX2.2	Subpallial GABA interneuron progenitor transcription factor
1.85	CALB1	GABA interneuron postmitotic phenotypic marker calbindin
1.82	GABRA5	GABA A receptor α subunit
1.75	GAD2	GABA synthesizing enzyme (GAD 65)
1.75	GABRB1	GABA A receptor β subunit
1.63	CALB2	GABA interneuron postmitotic phenotypic marker calretinin

A > P, anterior expression greater than posterior expression.

The subset chosen for investigation include a number of transcription factors. *Ascl1* (achaete-scute complex like homolog 1, also known as *Mash1*) is expressed in ventral telencephalon and specific areas of the diencephalon in rodents where it promotes differentiation of GABAergic interneurons (Casarosa et al. 1999; Horton et al. 1999; Yun et al. 2002). Letinic et al. (2002) interpreted evidence that *ASCL1* expression in progenitor cells and postmitotic GABAergic neurons of the dorsal pallium, in organotypic cultured explants of human forebrain from 13 weeks gestation, indicated that GABAergic neurons are born there rather than having migrated there. GABAergic interneurons were observed to migrate from subpallium to pallium in these explants but had ceased to express *ASCL1* on leaving the ventral proliferative zones. *Ascl1* acts together with *Dlx 1 and 2* (distal- less homeobox) in coordinating the differentiation of precursors of GABAergic interneurons by regulating Notch signaling, *Ascl1* being expressed earlier in development and *Dlx1/2* later (Yun et al. 2002). *Dlx5* and *6* are expressed in both developing and mature inhibitory interneurons, and may be necessary for the migration and differentiation, but not production, of interneurons born in the GE. Furthermore, they appear to be particularly important in specifying differentiation of parvalbumin-expressing basket cells (Wang, Dye et al. 2010).

In rodents, *Lhx6 is* a transcription factor expressed in the MGE and is required for the specification of parvalbumin- and somatostatin-positive interneurons in the neocortex and the hippocampus. It is required for the normal tangential and radial migration of GABAergic interneurons in the cortex (Liodis et al. 2007). However, LHX6 expression has been detected in the human cortical proliferative zones at 8 PCW (Jakovcevski et al. 2011). Similarly, *Olig2* is a marker for the proliferative zones of subpallial structures in developing rodent forebrain, showing an inverse pattern of expression with *Pax6*, the cortical progenitor marker (Heins et al. 2002). *Olig2* expressing progenitors can give rise to neurons early in development, including GABAergic neurons in the GE and oligodendrocytes at later stages (Takebayashi et al. 2000; Miyoshi et al. 2007). However, it is already known that, in human, the distinction is less clear cut, with numerous cells in cortical VZ/SVZ and in the GE at mid-gestation co-expressing both PAX6 and OLIG2 (Mo and Zecevic 2008).

We also studied the isoforms of the GABA synthesizing enzyme, glutamate decarboxylase (GAD) 67 and 65 kiloDaltons, coded for by the genes *GAD1* and *GAD2* (Erlander et al. 1991) and CALB2 (calretinin), a calcium binding protein expressed early in development by a subset of GABAergic neurons (Fonseca et al. 1995; Ulfig 2002) as well as genes coding for 3 subunits of the GABA-A ionotropic receptor, *GABRA5, GABRB1*, and *GABRB3* (Olsen and Sieghart 2008) all potentially more highly expressed anteriorly than posteriorly (see Table 1). The establishment of cortical neurogenesis of GABAergic interneurons in human at these stages of development, including localization of this phenomenon to more anterior regions of the cortical wall, adds greatly to our understanding of the difference between primate and rodent cortical development.

## Materials and Methods

### Human Embryonic and Fetal Brains

Human embryonic and fetal tissue was obtained from the joint MRC/Wellcome Trust-funded Human Developmental Biology Resource (HDBR, http://www.hdbr.org [date last accessed; 6 September 2013]) with appropriate maternal written consent and approval from the Newcastle and North Tyneside NHS Health Authority Joint Ethics Committee. HDBR is regulated by the UK Human Tissue Authority (HTA; www.hta.gov.uk) and operates in accordance with the relevant HTA Codes of Practice. Fetal staging was estimated from modified Carnegie Staging criteria (6.5–8 PCW, Bullen and Wilson 1997) or foot and crown-rump lengths (9–15 PCW) (Hern 1984). Fetal samples from ages 6.5–15 PCW were used for IHC and ISH (*n* = 43), and ages 8–12 PCW were used for qPCR (*n* = 8). Prior to sectioning, brains were fixed for at least 24 h at 4 °C in 0.1 M phosphate-buffered saline (PBS) containing 4% paraformaldehyde (PFA; Sigma Aldrich, Poole, UK). Whole or half brains (divided sagittally) were transferred to 70% ethanol for storage at 4 °C prior to paraffin embedding. Thick sections (8 µm) were usually cut sagittally, but sometimes coronally or horizontally, mounted on slides, and used for tissue ISH and IHC. For the immunofluorescence study, the frontal cortex from an 11 PCW fetus was fixed, cryoprotected in 30% sucrose in PBS, and sectioned with a cryostat; 20 µm sections were collected directly onto slides.

### RNA Isolation and Reverse Transcription

As previously described (Ip et al. 2010), right-sided cortices were dissected; subcortical structures, temporal lobes, and meninges removed; and 5 mm slices cut along the anterior–posterior axis. Total RNA was isolated from the anterior-most and posterior-most slices using the PeqGOLD RNAPure reagent (Peqlab, Fareham, UK) according to the manufacturer's instructions. The cDNA templates were synthesized by reverse transcription first-strand synthesis reaction from the extracted RNA using random hexadeoxynucleotide primers (Promega, Southampton, UK) and SuperscriptTM III Reverse Transcriptase Kit (Invitrogen, Paisley, UK) following the manufacturer's instructions for 2 μg of total RNA in a final volume of 50 μL. The transcribed cDNA template was further diluted 2-fold prior to the application of qPCR.

### Quantitative Real-Time PCR

The expression levels of 12 GABAergic interneuron-related genes (*ASCL1, CALB2, DLX1, DLX2, DLX5, GABRA5, GABRB1, GABRB3, GAD1, GAD2, LHX6,* and *OLIG2*) and 3 reference (housekeeping) genes (*β-ACTIN, GAPDH*, and *SDHA*) (Vandesompele et al. 2002) in the anterior-most and posterior-slices of neocortex (see above) were measured by qPCR. Primers were designed according to the standard criteria for PCR primer design using the Primer3 (v. 0.4.0) designing program (http://frodo.wi.mit.edu/primer3/ [date last accessed; 6 September 2013]). Primers synthesis and product sequencing for quality control were performed by Eurofins MWG Operon© (http://www.eurofinsgenomics.eu [date last accessed; 6 September 2013]). Table 2 shows the sequence and product size of each primer used. A SYBR Green-based rtPCR assay was performed in 7900HT Fast Real-Time PCR system (Applied Biosystems, Warrington, UK). A total volume of 10 μL qPCR reaction was set up in triplicates, containing 5 μL of 2 × SYBR Green qPCR Master Mix (Invitrogen, Paisley, UK), 1 μL of the diluted cDNA template, 0.5 μL of each primer (10 ρmol/μL), and 3 μL of Molecular Biology grade water. A negative control was incorporated by replacing the cDNA template with Molecular Biology grade water (VWR International, Lutterworth, UK). A standard thermal cycle protocol was used as previously described (Ip et al. 2010). The results of each reaction were analyzed by uploading raw data to the Real-Time PCR Miner web site (http://www.miner.ewindup.info [date last accessed; 6 September 2013]). This calculated the reaction efficiency and the fractional cycle number at threshold (CT) for each reaction (Zhao and Fernald 2005).

**Table 2:** Primers sets used in rtPCR experiment

Primers	Forward primer^a^	Reverse primer^a^	Product size (bp)
ASCL1	TGCACTCCAATCATTCACG	GTGCGTGTTAGAGGTGATGG	146
Β-ACTIN^b^	CTACAATGAGCTGCGTGTGGC	CAGGTCCAGACGCAGGATGGC	271
CALB2	GCTCCAGGAATACACCCAAA	CAGCTCATGCTCGTCAATGT	208
DLX1	TGTCTCCTTCTCCCATGTCC	ACTGATGTAGGGGCTGGATG	167
DLX2	GCACATGGGTTCCTACCAGT	TCCTTCTCAGGCTCGTTGTT	153
DLX5	ACCAACCAGCCAGAGAAAGA	GCAAGGCGAGGTACTGAGTC	151
GAPDH^b^	TGCACCACCAACTGCTTAGC	GGCATGGACTGTGGTCATGAG	86
FGFR3	CCACTGTCTGGGTCAAGGAT	TGTGTCCACACCTGTGTCCT	233
GABRA5	ATCTTGGATGGGCTCTTGG	TGTACTCCATTTCCGTGTCG	130
GABRB1	GTTTGTGTTCCTGGCTCTGC	GGCACTCTGGTCTTGTTTGC	100
GABRB3	CAGCATCGACATGGTTTCC	TCGATTGTCAAGCGTGAGG	124
GAD1	AGGCAATCCTCCAAGAACC	TGAAAGTCCAGCACCTTGG	218
GAD2	CGCATGGTCATCTCAAACC	AGTGGAACAGCTTGGTGAGC	114
LHX6	ACAGATCTACGCCAGCGACT	CATGGTGTCGTAGTGGATGC	157
OLIG2	GCTGCGTCTCAAGATCAACA	CACCAGTCGCTTCATCTCCT	196
SDHA^b^	TGGGAACAAGAGGGCATCTG	CCACCACTGCATCAAATTCATG	84

a All sequences are in a 5′–3′ direction.

b Reference genes.

### Quantitative Analysis of RNA Expression by Gene Chip, RNAseq, and qPCR

In order to verify our previous observations obtained by microarray (Ip et al. 2010), we compared these data with previously published RNAseq data (http://brainspan.org/rnaseq/search/index.html) and with qPCR data collected in the present study which was all converted to expression relative to the geometric mean expression of 3 reference genes (see above). A paired Student's *t*-test was used to determine the significance of differences between anterior and posterior expression. To make the 3 studies comparable, we calculated expression levels for all genes of interest relative to expression of the same reference genes.

Microarray data were obtained using the Human Genome U133 Plus 2.0 Array (Affymetrix Ltd., High Wycombe, UK) as previously described (Ip et al. 2010). GeneSpring GX7.3 software (Agilent Technologies, South Queensferry, UK) was used to undertake expression analysis. Expression for each probe was normalized by GC-RMA, and using the most highly expressed probe set for each gene of interest, the relative expression of each gene was calculated by dividing its expression level by the average expression of the 3 reference genes for each sample. Mean relative expression over the 6 samples, between 8 and 12.5 PCW of age, was calculated along with 95% confidence limits. A paired Student's *t*-test was used determine the significance of differences between anterior and posterior expression.

RNA seq data, obtained from RNA collected as part of the study of (Kang et al. 2011) was accessed via the database at http://brainspan.org/rnaseq/search/index.html. Anterior pole data were derived from 7 samples from 4 fetuses, the dorsolateral and ventrolateral prefrontal cortex at 8, 9, and 12 PCW (2 fetuses at 12 PCW, no data available for the dorsolateral prefrontal cortex at 9 PCW). Posterior pole data were taken from 4 samples from the same 4 fetuses labeled occipital cortex at 8 and 9 PCW and primary visual cortex at 12 PCW. The relative expression of each gene was calculated by dividing its expression level by the average expression of the 3 reference genes for each sample. Mean relative expression of the 7 anterior samples and 4 posterior samples, between 8 and 12 PCW of age, was calculated along with 95% confidence limits. An unpaired Student's *t*-test was used determine the significance of differences between anterior and posterior samples.

### Manufacture of TISH Probes

Total RNAs from human fetal whole brain or neocortex aged between 8 and 12 PCW were isolated with Trizol (Invitrogen) according to the manufacturer's instructions and reverse transcribed into cDNA templates. Gene-specific primer sets (Eurofins MWG Operon) were designed using Primer 3 program (http://frodo.wi.mit.edu/primer3/) and were incorporated with either SP6 (forward) or T7 (reverse) consensus sequences. The primer sequences and amplicon size for each probe are listed in Table 3. Procedures and conditions for PCR, and subsequent gel extraction, in vitro transcription Digoxigenin (DIG)-labeling reaction, labeled probes purification, and quality controls were carried out as previously described (Bayatti, Sarma et al. 2008).

**Table 3:** TISH probes

Probe	Forward primer (SP6)*	Reverse primer (T7)*	Product size (bp)
DLX1	AATACGATTTAGGTGACACTATAGAATACGCACTACTCCATGCACTGTTTAC	TAAGTTAATACGACTCACTATAGGGCGATGCTTCATCAGCTTCTTGAACTT	453
DLX2	AATACGATTTAGGTGACACTATAGAATACACAGCAGCTACTACACCAACCAG	TAAGTTAATACGACTCACTATAGGGCGAACCACTTTTCCACATCTTCTTGA	455
DLX5	AATACGATTTAGGTGACACTATAGAATACTACGCTAGCTCCTACCACCAGTA	TAAGTTAATACGACTCACTATAGGGCGACTTGTGTACCAGGATGCAGAGTT	497
GAD1	AATACGATTTAGGTGACACTATAGAATACGGATTGGATATTATTGGCCTAGC	TAAGTTAATACGACTCACTATAGGGCGATCAAAAGCTCCATAAACAGTCGT	482

### Tissue in Situ Hybridization

ISH was performed as previously described (Bayatti, Sarma et al. 2008) with some modifications. Paraffin sections were de-waxed and rehydrated before being incubated with proteinase K (20 μg/mL; Sigma-Aldrich) for 8 min at room temperature. Sections were fixed in 4% PFA/PBS for 20 min, washed in PBS, and treated with 0.1 M triethanolamine (Sigma-Aldrich, pH 8.0)/0.25% acetic anhydride (Sigma-Aldrich)/0.2% HCl for 10 min, dehydrated in ethanol and air-dried. DIG-labeled probes (300 ng) were used per 100 μL of DIG Easy Hyb mixture (Roche, Lewes, UK). Probe/Hyb mix (200 μL) was used per slide, covered with glass coverslips. Slides were incubated in a hybridization chamber overnight at 68 °C, rinsed in 5× standard sodium citrate (SSC, pH 7.2) at 65 °C to remove coverslips, followed by 3 washes at 50 °C (2 × SSC twice and 0.2 × SSC once), followed by 1 wash with 0.2 × SSC once at room temperature. After briefly rinsing in 0.1 M Tris (pH 7.6)/0.15 M NaCl (Buffer 1) and blocking with 10% fetal calf serum (Invitrogen)/Buffer 1 for 1 h at room temperature, sections were incubated with anti-DIG antibody (Roche; diluted 1:1000 in 2% FCS/Buffer 1) overnight at 4 °C. Sections were washed in Buffer 1 for 6 × 30 min. Detection of probes/anti-DIG antibody was achieved by addition of NBT/BCIP solution (Roche; 20 μL/mL) in 0.1 M Tris (pH 9.5)/0.1 M NaCl (Buffer 2). The color reaction was developed in the dark for several hours to overnight and terminated by rinsing slides in Buffer 2 and then distilled water. Sections were mounted in Aquamount. Comparison of staining between sense and antisense probes was carried out to ensure specificity (see Supplementary Fig. 1).

### Immunohistochemistry and Immunofluorescence

Paraffin sections of forebrain were immunoperoxidase stained on slides, according to standard protocols as previously described (Bayatti, Moss et al. 2008). Details of the sources of primary antibodies used and working dilutions are provided in Table 4. Biotinylated secondary antibodies and streptavidin-HRP conjugates were obtained from Vector Labs (Peterborough, UK). Ready-made diaminobenzidine solution was obtained from Invitrogen.

**Table 4:** Primary antibodies used in immunohistochemical studies

Primary antibody	Manufacturer	Catalog no.	Species	Dilution used
Anti-MASH1/Achaete-scute homolog 1 antibody	Abcam; Cambridge, UK	ab74065	Rabbit polyclonal	1:400
Anti-calretinin	Swant; Marly, Switzerland	6B3	Mouse monoclonal	1:1000
Anti-calretinin	Abcam; Cambridge, UK	ab702	Rabbit polyclonal	1:100
Anti-Dlx2 [4B9]	Abcam; Cambridge, UK	ab117546	Mouse monoclonal	1:400
Anti-Dlx2: neural stem cell marker	Abcam; Cambridge, UK	ab18188	Rabbit polyclonal	1:200
Anti-GAD65 [GAD-6]: neuronal marker	Abcam: Cambridge, UK	ab26113	Mouse monoclonal	1:500
Anti-Ki67 clone MIB-1	Dako; Cambridge, UK	IR626	Mouse monoclonal	1:400

Frozen-fixed sections were immunofluorescently stained on slide. Sections were incubated with a mixture of 2 primary antibodies at the appropriate concentration (see Table 4) and the appropriate serum (3% v/v) in PBS and 0.1% Triton x-100 (Sigma-Aldrich) overnight at 4 °C. Sections were washed and then incubated with fluorescently conjugated secondary antibodies (Alexa Fluor^®^ 594 donkey conjugated anti-mouse, and Alexa Fluor^®^ 488 donkey conjugated anti-rabbit obtained from Molecular Probes^®^, Paisley, UK) for 2 h at room temperature prior to washing and mounting in VectorShield (Vector labs).

### Optical Densitometry

Quantification of density of Immunostaining for ASCL1, DLX2, GAD2, and CALB2 was performed to confirm their tangential expression gradients, using the ImageJ^®^ 1.42 h software (NIH; http://rsbweb.nih.gov/ij/ [date last accessed; 6 September 2013]). Photographs were taken from the anterior- and posterior-most extents of sagittal sections at 8, 9, 10, and 12 PCW for comparison (at least 2, usually 3, fetal brains were examined at each age and 1 or 2 sections from each brain were used for statistical analysis). All photographs were taken with the same exposure time and cropped to similar widths. The average optical density of histological staining in the CP and VZ was measured in 3 rectangular boxes of equal widths for each section anteriorly and posteriorly, which spanned the thickness of the CP, or the VZ, and were placed adjacently. To take into account of background staining, the ratio of mean gray values anterior to posterior in CP and VZ was calculated.

## Results

### GABAergic Gene Expression in the Early Fetal Neocortex

**Figure 1.**
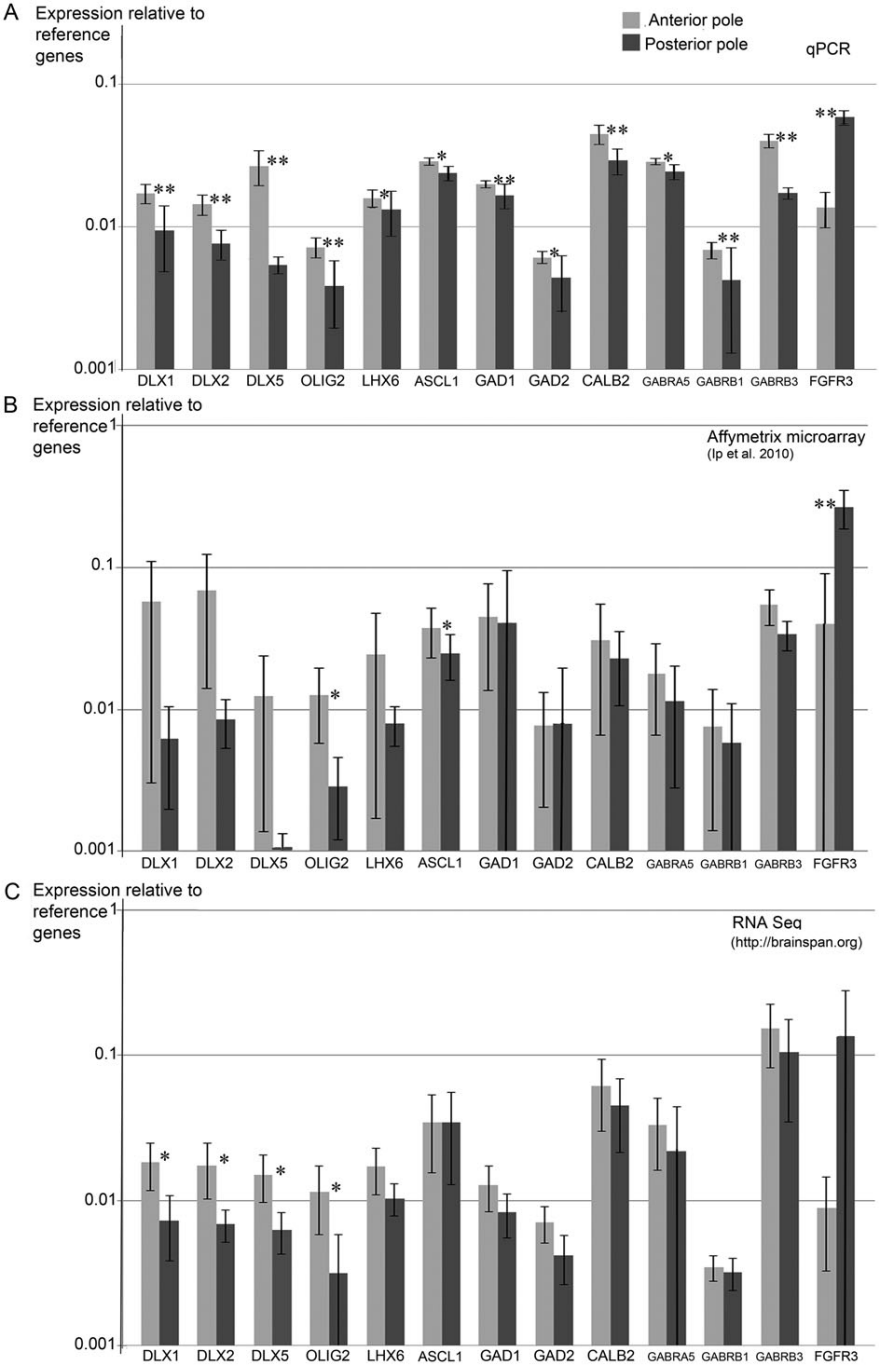
qPCR confirmation of gradients of GABAergic gene expression between 8 and 12 PCW, and comparison with microarray and RNA seq data from previous studies. The mean expression of the genes of interest, relative to the average expression of 3 reference genes, *β-ACTIN, GAPDH*, and *SDHA*, from RNA samples taken from the anterior and posterior poles of the human neocortex between 8 and 12 PCW, is shown for qPCR data collected in the present study (*A*, *n* = 8 fetuses) Affymetrix microarray (*B*, *n* = 6, Ip et al. 2010) and RNA seq (*C*, *n* = 4, http://brainspan.org/rnaseq/search/index.html). The general patterns of expression are the same for all 3 studies, although the qPCR study shows less experimental variability and thus detected differences between the anterior and posterior poles with greater confidence. Clear evidence is provided for higher anterior expression of all GABAergic genes at this time. *FGFR3* expression is included as an example of a posteriorly expressed gene. **P < 0.01, *P < 0.05, error bars represent 95% confidence limits. Note that the chart is plotted on a logarithmic scale resulting in asymmetric error bars.

A qPCR study was carried out to validate and extend the results obtained from our previous microarray study (Ip et al. 2010). In addition, we accessed publically available RNA seq data (http://brainspan.org/rnaseq/search/index.html) at the relevant developmental time points, and expressed all 3 datasets in a way that makes them comparable (Fig. 1). All 3 approaches gave broadly similar patterns of gene expression with nearly all GABAergic genes selected for study showing higher expression at the anterior compared with the posterior pole. However, this only reached statistical significance for all markers in the qPCR study. This validates the approach of collecting global gene expression data by microarray or whole RNA sequencing as a way of discovering potentially interesting patterns of gene expression. Nevertheless, confirmation by qPCR and other methods are still required.

The known posterior marker *FGFR3* (O'Leary and Nakagawa 2002; Iwata and Hevner 2009) was more highly expressed at the posterior pole in all 3 studies, showing that consistently higher expression of the genes of interest at the anterior pole was not some methodological anomaly. *DLX5* showed the consistently largest difference anterior to posterior. All transcription factors showed broadly similar levels of expression, although expression of *ASCL1* was consistently high and was the least regionalized, whereas *OLIG2* usually showed the lowest expression. Of the 2 glutamate decarboxylase isoforms, *GAD1* was generally more highly expressed than *GAD2. CALB2* generally showed higher expression than the *GAD* genes suggesting it may be expressed by cells other than GABAergic interneurons. Of the GABA receptor subunit genes studied, *GABRB3* and *GABRA5* exhibited some of the highest expression levels of all transcripts studied, whereas *GABRB1* showed low expression.

**Figure 2.**
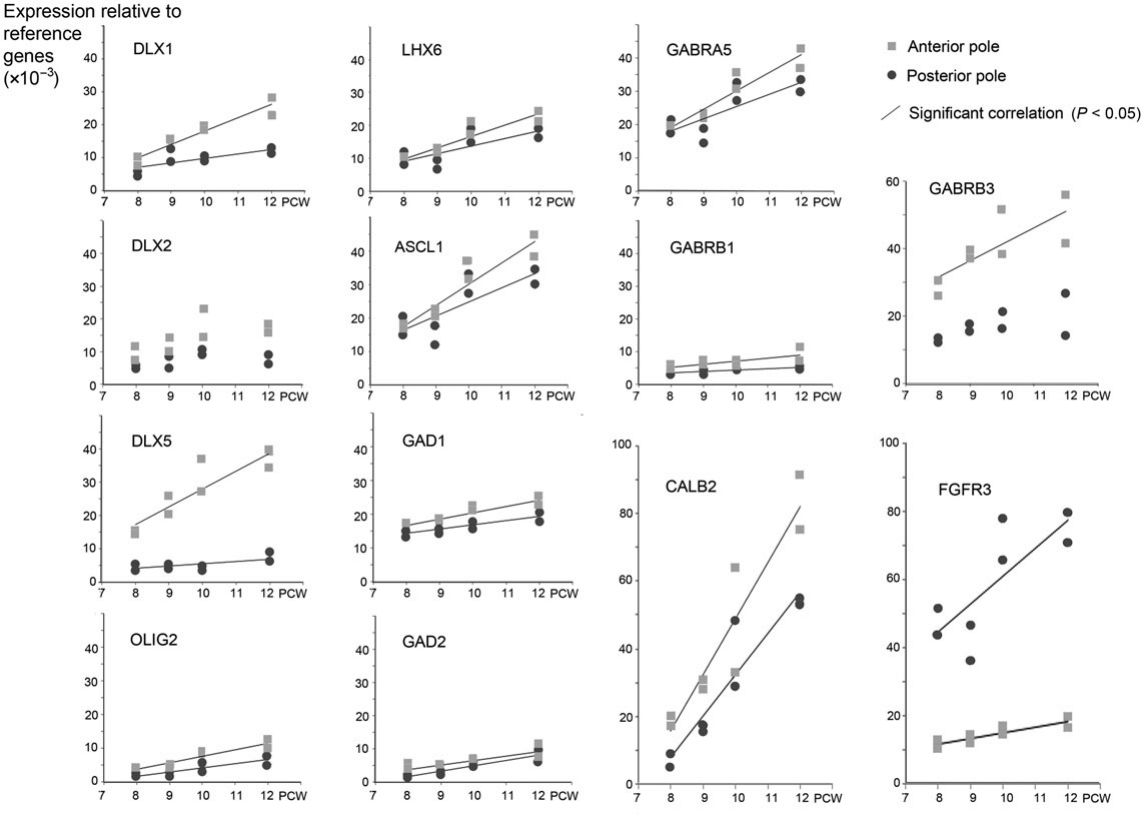
Temporal changes in GABAergic gene expression by qPCR between 8 and 12 PCW. The expression of the genes of interest, relative to the average expression of 3 reference genes, from RNA samples taken from the anterior and posterior poles of the human neocortex between 8 and 12 PCW, is shown for qPCR data collected in the present study (*n* = 8 fetuses). Statistically significant linear correlations of expression over time (*P* < 0.05) are marked with a line. It can be seen that nearly all genes showed increased expression, both anteriorly and posteriorly, with age, although for some genes, notably *DLX5* and *GABRB3*, expression increased more quickly anteriorly, compared with posteriorly. *FGFR3* expression is included as an example of a posteriorly expressed gene.

**Figure 3.**
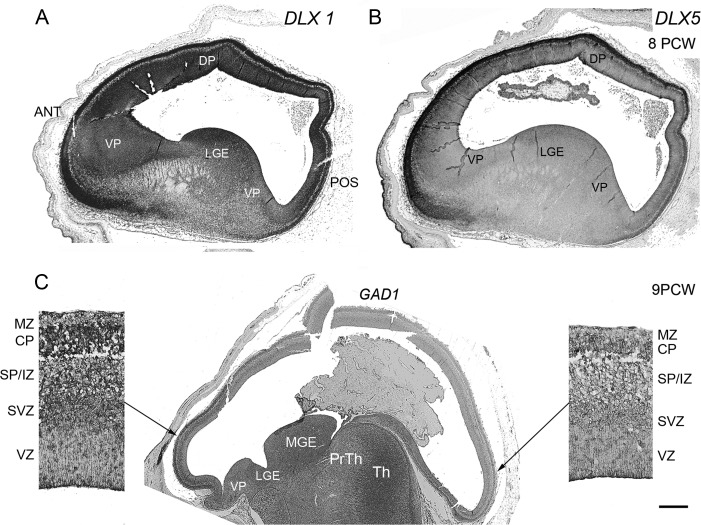
Expression gradients for GABAergic genes shown by in situ hybridization. Examples of *DLX* expression are shown in lateral sagittal sections at 8 PCW (*A*,*B*). There was moderate expression in the lateral ganglionic eminence (LGE) but weakest expression at the boundaries between ventral pallium (VP) and LGE before moderate expression resumes in the VZ and SVZ, and strong expression was observed in the cortical plate (CP) of the VP and dorsal pallium (DP). A gradient of expression from anterior (Ant) to posterior (Pos) poles of the cortex was also discernible. GAD1 expression was shown at 9 PCW in a medial section (*C*). In subcortical structures, expression was relatively strong in the lateral and medial (MGE) ganglionic eminences, and in the thalamus (Th) but weak in the prethalamus (PrTh). There was less expression at the boundary of the LGE with the cortex (ventral pallium, VP) but relatively high expression in the anterior cortical wall compared with the posterior. Higher magnification images show GAD1 expression throughout the anterior cortical wall but highest in the SVZ and CP where many, but not all, cells appear stained. In the early subplate and intermediate zone (SP/IZ), an area of low cellular density, heavily stained cells are discernible, while in the VZ only a few moderately stained cells can be seen. In the posterior cortical wall, the pattern was similar although staining was less intense. Scale bar represents 100 μm in high-magnification images and 800 μm at low magnification. The orientation of all the section images are the same, hence Ant and Pos are the same as indicated in *A*.

Consistent patterns of expression over time were also observed (Fig. 2). Nearly all genes showed increasing expression with age. In most cases, anterior and posterior expression levels increased in parallel, although anterior was consistently higher than posterior. For some genes, notably *DLX1, DLX5,* and *GABRB3*, expression at the anterior pole increased at a faster rate than at the posterior pole. ISH studies confirmed the expression of 4 of these genes, *DLX1, 2,* and *5* and *GAD1*, and all showed gradients of expression from anterior to posterior poles at 8 and 9 PCW (Fig. 3, *DLX2* not shown). Although expression of these genes was detected by ISH in the GE, as would be expected from animal studies, expression was higher in the neocortex, particularly at the anterior pole.

### Demonstration of Gradients of Expression by Immunohistochemistry

**Figure 4.**
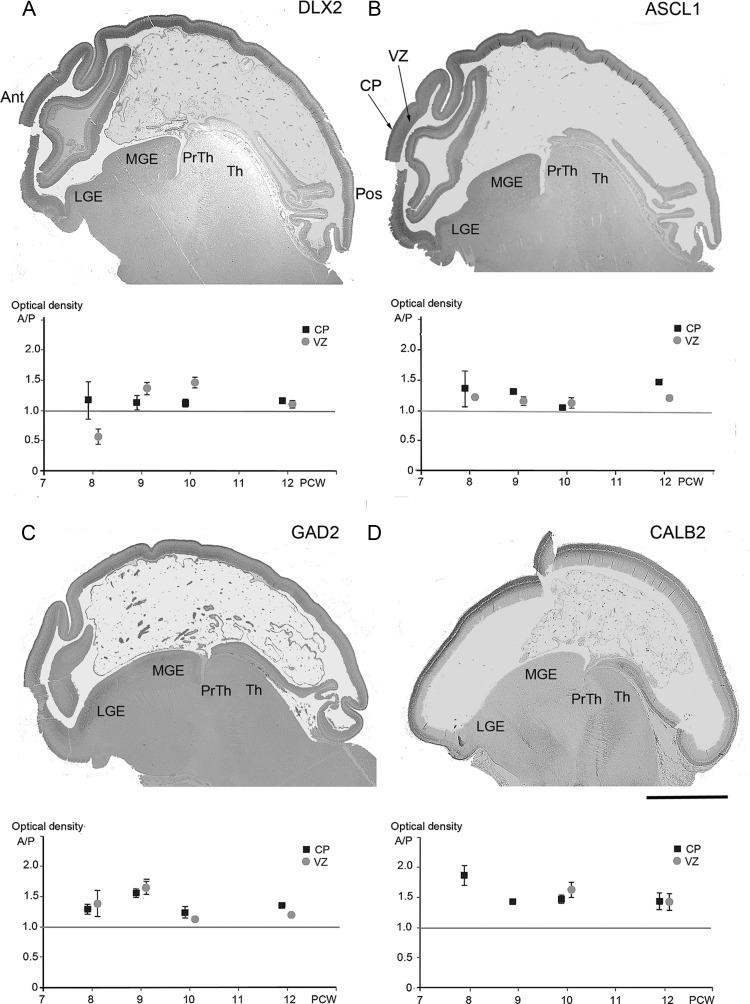
Expression gradients for immunoreactivity to GABAergic markers confirmed by optical densitometry. IHC for four proteins was selected for measurement at multiple ages; DLX2 (*A*), ASCL1 (*B*), GAD2 (*C*), and CALB2 (*D*). Low power images of immunostaining for each protein are shown, all at 9 PCW in medial sagittal sections. DLX2, ASCL1, and GAD2 all show moderate immunoreactivity in the medial ganglionic eminence but stronger expression in the neocortex, especially in the cortical plate (CP) but also in the ventricular and subventricular zone (VZ/SVZ) and a gradient of expression from the anterior to posterior pole of the neocortex. CALB2 also shows a gradient of expression, but differs from the other 3 in showing moderate expression in fibers in the subplate and intermediate zone (SP/IZ) but low expression in VZ/SVZ and the MGE. The ratio of anterior to posterior labeling density was calculated for each section and the mean plotted. Error bars represent 95% confidence limits of the mean, which are sometimes close enough together to be obscured by the representative symbol. With the exception of DLX2 expression at 8 PCW, there was consistently higher expression anteriorly for all genes and time point studied. The prethalamus (PrTh) and thalamus (Th) show low immunoreactivity for all four markers, with the exception of CALB2 expression in the thalamus. Scale bar = 2 mm. The orientation of all the section images is the same, hence Ant and Pos are the same as indicated in *A*.

**Figure 5.**
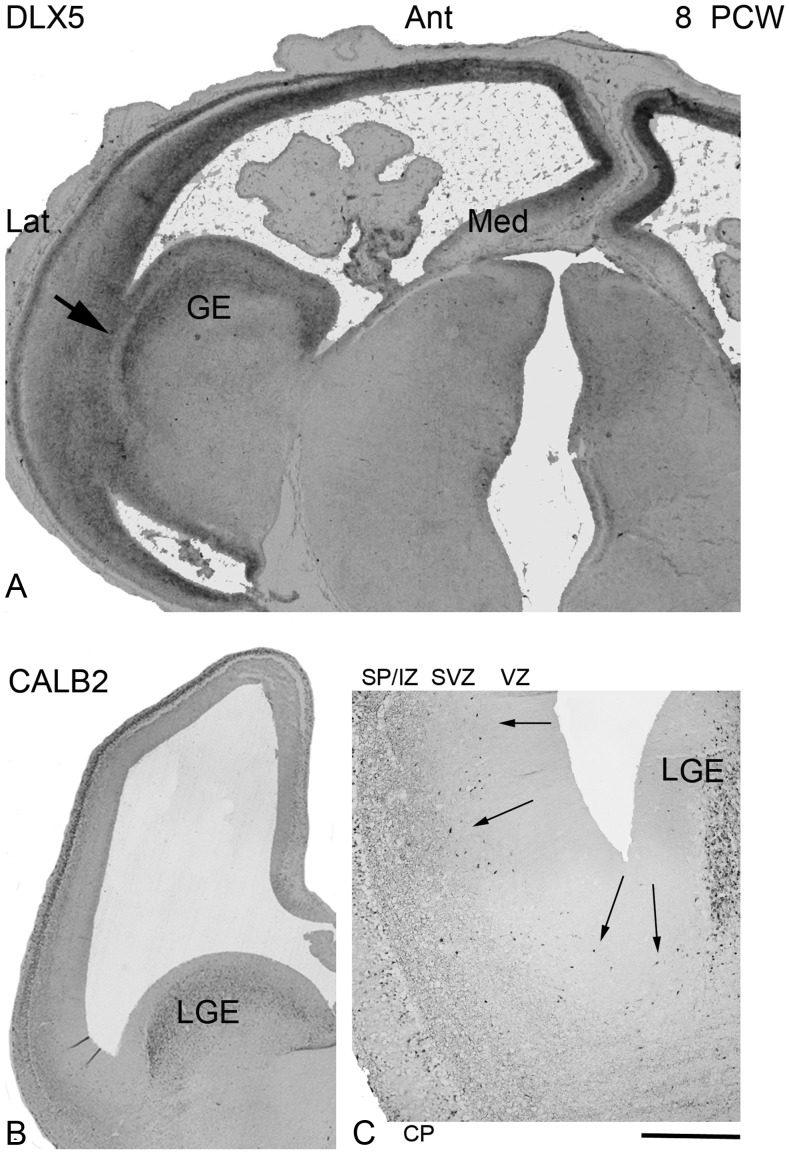
Expression of GABAergic genes in horizontal sections at 8 PCW. Staining patterns for ISH for *DLX5* (*A*), and IHC for CALB2 (*B*) in horizontal sections at 8 PCWs serve to further illustrate that there were 2 centers of strongest expression, in the ganglionic eminence, and in the dorsal pallium, particularly at more anterior (Ant) locations, with no evidence of a gradient of expression between the sub pallium and dorsal pallium that would suggest a migratory stream of cells moving between the 2. Indeed, there appeared to be distinct break in DLX5 expression between the postmitotic layers of the ganglionic eminence (GE) and cortical plate of the ventral pallium (arrow). However, at higher magnification, a few CALB2-positive neurons appeared to be crossing this zone (*C*, small arrows). Scale bar = 100 μm in *A*,*B*; 300 μm in *C*. The orientation of all the section images are the same, hence Ant is the same in *B* indicated in *A* (*C* is image of section in *B* at higher magnification).

Whole sagittal sections of fetal brain were also successfully immunostained for 4 markers of GABAergic interneurons, DLX2, ASCL1, GAD2, and CALB2 between 8 and 12 PCW weeks. At all stages, a similar expression pattern was observed for DLX2, ASCL1, and GAD2, with moderate expression in the GE, but stronger expression in the CP (Fig. 4). There was also some expression in the VZ/SVZ when compared with areas of completely negative staining in the diencephalon. CALB2 showed a different pattern of staining. CALB2-positive neurons were present in the postmitotic layers of the lateral (Fig. 5*B*,*C*) and caudal (not shown) GE, but were absent from the MGE (Fig. 4*D*). In the cortex, CALB2-positive neuronal cell bodies were prominent in the cortical plate (CP) at 8–9 PCW, with fewer also present in the marginal zone and subplate, but with many immunoreactive fibers present in the subplate and intermediate zone (Figs 4*D* and 5*B*). No immunostaining was present in the proliferative zones at these earlier stages.

In many sections, a clear gradient of expression between the anterior and posterior poles of the neocortex was discernible at low magnification (Fig. 4). Optical density measurements of immunostaining in the CP and ventricular zone (VZ) confirmed a consistently higher density of staining in the anterior compared with the posterior pole in nearly all sections studied, the notable exception being DLX2 expression at 8 PCW (Fig. 4*A*). The conclusion from these studies is that there was a higher density of GABAergic neurons and their precursors in the anterior pole of the neocortex but a more detailed study of expression patterns was required to discern whether these neurons are generated in the cortex, or migrate there from subcortical structures at this stage of development.

### Cellular and Laminar Localization of Expression of GABAergic Markers in the Forebrain

**Figure 6.**
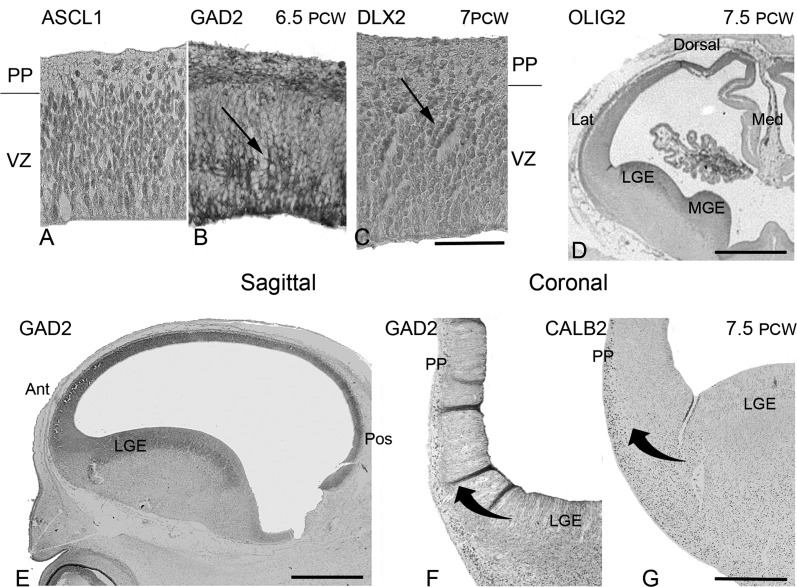
Expression of GABAergic genes prior to cortical plate formation. Immunoreactivity for ASCL1 (*A*) GAD2 (*B*) and DLX2 (*C*) a little later was detectable both in cells of the proliferative ventricular zone (VZ) and postmitotic preplate (PP) confirming GABAergic neurons are born in the cortex at this early stage. Arrows point to radially orientated aggregates of cells expressing GAD2 and DLX2 suggesting these cells were being born in the ventricular zone and migrating radially. Low power images confirm that both OLIG2 (*D*, coronal section) and GAD2 (*E*, sagittal section) were as strongly expressed in the anterior/dorsal regions of the dorsal pallium as in the ganglionic eminences at this stage, although at higher magnification in coronal sections, it was possible to see a prominent tangential stream of cells (curved arrow) either expressing GAD2 (*F*) or CALB2 (*G*) apparently migrating from the ganglionic eminence toward the preplate. Scale bars = 50 μm in *A*, *B*, *C*; 500 μm in *F*, *G*; and 2 mm in *D*, *E*.

**Figure 7.**
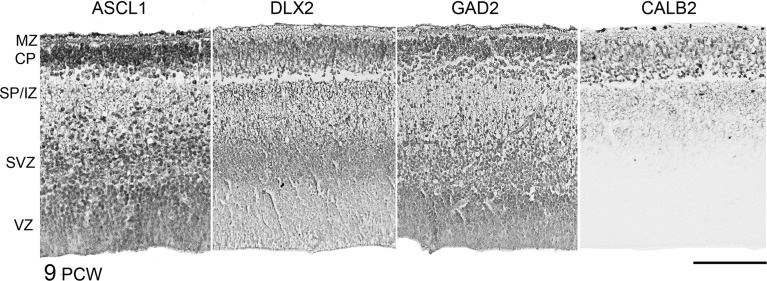
Laminar expression of GABAergic immunoreactivity at 9 PCW. ASCL1, DLX2, and GAD2 show similar patterns of immunoreactivity at 9 PCW, with a mosaic of cells exhibiting either strong, moderate, and weak/no expression throughout the layers of the cortical wall. They differed in that ASCL1 expression was generally more prominent in the VZ, whereas DLX2 was stronger in the SVZ. GAD2 immunoreactivity was observed in proliferative layers as well as in postmitotic cells. In the cortical plate (CP), most GABAergic markers showed more expression in cells closer to the outer boundary with the marginal zone. The exception was CALB2 immunoreactivity, which was most prominent in cells at the interface between the CP and the early subplate (SP/IZ). CALB2-positive fibrers were also seen extending into the intermediate zone (IZ), but no CALB2-positive cells were seen in the SVZ/VZ at this stage. Scale bar = 200 μm.

At 6.5–7.5 PCW, we confirmed the presence of GAD2 and CALB2 expressing neurons in the preplate as previously described (Meyer et al. 2000; Rakic and Zecevic 2003). In addition, DLX2 and ASCL1 immunoreactivity was observed. For all markers, positive cells were seen in VZ, sometimes in radially arranged clusters (Fig. 6). ASCL1 immunoreactivity was more prevalent in the VZ, whereas the others showed stronger expression in the preplate. This fits with the idea that ASCL1 is expressed earlier in the development of interneurons (see Introduction section) and strongly suggests that GABAergic cells are born in the cortical wall at this stage of development. ASCL1, DLX2, and GAD2 showed similar patterns of immunoreactivity between 8 and 12 PCW, with a mosaic of cells exhibiting either strong, moderate, and weak/no expression throughout the layers of the cortical wall (Fig. 7). They differ in that ASCL1 expression is generally more prominent in the VZ, whereas DLX2 was stronger in the SVZ. Despite being a marker for synaptic terminals in adult tissue (Erlander et al. 1991; Esclapez et al. 1994), GAD2 immunoreactivity was observed in proliferative layers (Fig. 6) as has been previously described (Petanjek et al. 2009). *GAD1* TISH staining was also most prominent in the SVZ and CP at 9 PCW (Fig. 3*C*). In the CP, most GABAergic markers showed more expression in cells closer to the outer boundary with the marginal zone. The exception was CALB2, immunoreactivity which was most prominent in cells at the interface between the CP and the early SP. This confirms the previous suggestion (Meyer et al. 2000) that these CALB2 cells are pioneer cells sending axons toward the thalamus rather than GABAergic cells. CALB2-positive axons can be observed in the early SP, intermediate zone, and into the internal capsule and CALB2 positive GABAergic neurons may exist at other locations in the CP and early SP.

**Figure 8.**
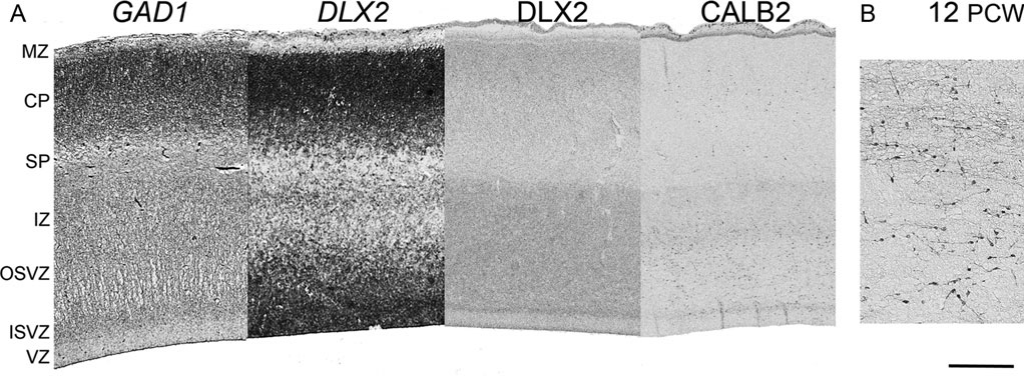
Laminar expression of GABAergic genes at 12 PCW. Panel (*A*) shows at low magnification the expression of *GAD1* and *DLX2* by ISH, and DLX2 and CALB2 by IHC. *GAD1* expression indicated the presence of GABAergic neurons in the cortical plate at this stage, and the expression of *DLX2*/DLX2 in the proliferative zones suggests that GABAergic cells may have been generated intracortically at this stage. This stage of development is noticeable for the appearance of CALB2 immunoreactive neurons in the VZ, SVZ, and intermediate zones. At higher magnification (*B*), many of these CALB2-positive cells appeared migratory, but have a mix of orientations suggestive of tangential or radial migration. Note that the marginal zone (MZ) has a cell-containing outer layer (subpial granular layer) at this age. Scale bar = 500 μm in *A*; 100 μm in *B*.

**Figure 9.**
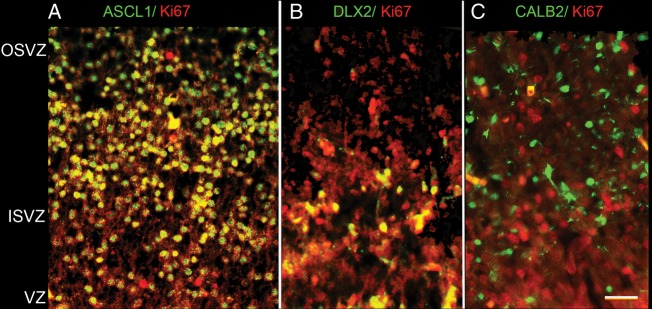
Double labeling of GABAergic interneuron precursors with the cell-division marker Ki67. Ki67 immunofluorescence (red) was observed in many cell nuclei throughout the proliferative layers of the neocortex. This coincided with expression of the transcription factors ASCL1 and DLX2 (green) and a majority of cells appeared double labeled for Ki67 and either one of the transcription factors (yellow, 9*A*,*B*). DLX2 was less widely expressed than ASCL1, being largely confined to the ISVZ (*B*). CALB2 was also widely expressed in the SVZ (green, *C*) but was not seen to be co-expressed with Ki67. Scale bar = 100 μm.

By 12 PCW, as previously described (Bayatti, Moss et al. 2008), CALB2-positive neurons were now observed in the proliferative zones and intermediate zone, particularly at the anterior pole (Fig. 8). Many of these cells were of bipolar appearance, suggesting they are migratory cells. There was a mixture of radially and tangentially orientated cells, and alignments in between (Fig. 8*B*). At this stage, other GABAergic genes were also expressed, including *GAD1* and *DLX2* detected by ISH (Fig. 8*A*) and also DLX2 by IHC. In each case, expression was prominent in the upper CP and outer SVZ (OSVZ). DLX2 immunoreactivity was also prominent in the inner SVZ (ISVZ) but not the VZ, whereas *DLX2* mRNA expression was observed in the VZ (Fig. 8*A*). In order to confirm the expression of GABAergic markers in proliferating cells in the cortical wall, double immunofluorescent staining was carried out for the cell division marker Ki67 (Brown and Gatter 1990; Zecevic et al. 2011) and either ASCL1, DLX2, or CALB2 (Fig. 9). All 4 proteins were detected in the VZ and SVZ at 11 PCW, although DLX2 positive cells were mostly confined to the ISVZ. Only ASCL1 and DLX2 showed extensive double-labeling with Ki67; no CALB2 positive cells were found to be also Ki67 positive.

These observations point toward intracortical generation of GABAergic neurons but to what extent might subcortical interneurons invade the cortical wall at this stage of development? Expression of GABAergic markers was clearly seen in the GE. At stages up to 7.5 PCW, before formation of the CP, a prominent migratory stream of cells expressing some GABAergic markers such as GAD2 and CALB2 was seen extending from the GE toward the early subplate (Fig. 6*F*,*G*). However, even at these stages, GABAergic genes (e.g., GAD2 and OLIG2) showed expression in the CP that was stronger in dorsal and anterior regions of dorsal pallium than in the ventral pallium at the border with the GE (Fig. 6*D*,*E*). From 8 PCW onward, expression of GABAergic genes was not as strong in the GE as in the cortical wall. CALB2 immunoreactive cells were only present in lateral ganglionic eminence (LGE) and CGE, and not in the MGE (Fig. 4*D*) and only a very few CALB2-positive neurons appeared to be migrating from the GE into the early SP and intermediate zone of the dorsal pallium (Fig. 5*C*). For all markers, a continuous band of positive staining between the postmitotic zones of the GE and the strong labeling of the anterior cortical wall was not observed; rather, there was a break, or reduction, in expression at the boundary between the pallium and the subpallium (Figs 3 and 4). This was particularly apparent in the pattern of expression shown by *DLX5* in Figure 5*A*.

## Discussion

It is known that GABAergic neurons are present in the preplate, and there is strong evidence that at least some of these are generated intracortically (Zecevic et al. 1999; Meyer et al. 2000) supported by observations made in the present study. There is also good evidence that, around midgestation in humans, the cortical proliferative zones generate CALB2 positive interneurons in particular, which populate the outer layers of the cortex (Jakovcevski et al. 2011). However, what occurs during the developmentally important period between formation of the CP and innervation of the subplate by thalamic afferents (8–15 PCW) has not been studied in so much depth. In vitro experiments with cultured fetal tissue fragments (14/15 PCW) failed to find any evidence of generation of GABAergic neurons by cortical proliferative cells (Hansen et al. 2010) challenging the conclusion drawn from the first studies to explore this question (Letinic et al. 2002; Rakic and Zecevic. 2003) that interneurons were intracortically generated at this stage. A more recent study has found cells positive for the GABA neuron indicative transcription factors DLXs, NKx2.1, and LHX6 in the VZ and CP between 8 and 15 PCW (Jakovcevski et al. 2011) but in a limited number of samples (3) and the authors suggest that these may have migrated into the cortical wall from the GE. A study in macaque has suggested that GABAergic cells populating the early CP migrate there from the GE prior to a later wave of intracortical interneuron generation (Petanjek et al. 2009). It is conceivable that cells originating from the GE retain the ability to proliferate on reaching the cortical SVZ (Lui et al. 2011). We have now made an extensive study of GABAergic gene expression in the neocortex between 8 and 12 PCW and found robust evidence for expression of a wide range of genes associated with GABAergic neurogenesis and neurotransmission during the earliest stages of CP formation, confirming that the cortical cerebral wall is populated with GABAergic neurons and their precursors at this stage. But In addition, we have shown that there is a higher expression of these genes at the anterior pole of neocortex compared with the posterior pole, by qPCR, ISH, and IHC.

Our qPCR analysis showed that the expression of these genes, relative to reference genes, was generally found to increase between 8 and 12 PCW, suggesting that the GABAergic neurons are being added to in number, and undergoing differentiation, throughout this period. Although previous studies have shown that GABA synthesis is present at the earliest stages of neuronal specification (Del Rio et al. 1992; Zecevic and Milosevic. 1997) transcription factors involved in interneuron specification (*ASCL1,* the *DLXs, LHX6, OLIG2*) appear to be more highly expressed than the key markers of differentiation; *GAD1* and *GAD2*. This would indicate that many GABAergic neurons are new born and/or have not undergone extensive differentiation and this is supported by our histological observations that show that many of these genes and their proteins are expressed in the proliferative zones or in small, undifferentiated cells in the CP. Only a relatively small number of GABAergic cells in the subplate and marginal zone have extended dendrites and axonal arbors at this stage and are likely to be integrated into active neural circuitry (Moore et al. 2009; Wang, Hoerder-Suabedissen et al. 2010) whereas the tightly packed cells of the CP are very largely electrically inactive up to ages beyond those examined in this study (Moore et al. 2009). Furthermore, *ASCL1*, the earliest marker for interneuron specification (Yun et al. 2002) was the most highly expressed transcription factor studied, it was found at a higher level in the VZ than the others (Figs 6 and 7) and was extensively co-expressed with a cell proliferation marker (Fig. 9). This strongly suggests an intracortical origin for a high proportion of these GABAergic cells. However, it should be borne in mind that co-expression of *Ascl1* and *Olig2* in rodents can also specify an early population of oligodendrocyte precursors in the forebrain (Parras et al. 2007) although the oligodendrocyte precursor-specific marker PDGFR-α receptor is only expressed by a tiny proportion of cells in the human forebrain before 15 PCW at which time numbers begin to increase (Jakovcevski et al. 2009).

Other postdifferentiation markers, *CALB2*, and the *GABRA5* and *GABRB3* receptor subunits, were also more highly expressed than the *GADs*. This may be because these markers are not confined to GABAergic neurons. It is known that CALB2 is expressed by Cajal–Retzius cells in the marginal zone (Verney and Derer 1995; Meyer et al. 2000) and by pioneer neurons of the lower CP that extend axons toward the thalamus (Meyer et al. 1998, 2000). We have no evidence for expression of protein products of GABA-A receptor subunit genes and so the mRNA expression may be misleading, however, expressions levels could be higher because receptors can be expected to be localized to any cell that is a target for GABAergic neurotransmission, or even autocrine/paracrine transmission. It is well established in model systems that GABA is released tonically from immature cells that have yet to develop a vesicular mode of transmitter release (Demarque et al. 2002). There is evidence that signaling through GABA-A receptors might control proliferation in progenitor cells (Lo Turco et al. 1995; Haydar et al. 2000; Wang and Kriegstein 2009). Migrating neurons, of all phenotypes, in the cortex express GABA-A receptors, stimulation of which promotes migration away from the proliferative zones but stops migration in the appropriate cortical layer (Behar et al. 2000; Manent and Represa 2007). *Gabra5* has recently been identified as a SP marker in rodent where it is also expressed in an anterior to posterior gradient (Oeschger et al. 2012). *GABRB3*, which showed remarkably high expression, is implicated in autism-related disorders and neurodevelopmental epilepsy (Buxbaum et al. 2002; Kang and Barnes 2013). *GABRB1* showed relatively low expression, as is the case in the mature CNS (Olsen and Sieghart 2008). Further study of the cellular localization of these subunits in developing human brain is required and might provide important clues as to the function of GABA in cortical development and their implication in neurodevelopmental diseases.

### Why is There Higher Expression in the Anterior Neocortex?

Three possible mechanisms, alone or a combination, may explain higher GABAergic gene expression at the anterior cortical pole. First, the anterior cortex may mature more quickly than the posterior pole and express higher levels of markers of neuronal differentiation earlier. Second, there is extensive migration of GABAergic neurons and/or their precursors from the LGE/MGE into the anterior cortical wall at this stage of development. Third, the anterior neocortex is a preferred site for interneuron generation at this time.

Cortical development appears to unfold along an anterior/lateral to posterior/medial axis, judging by the thickness of the CP, which develops first in the lateral cerebral wall close to the future corticostriatal junction (Müller and O'Rahilly 1990; Zecevic 1993). But this is a simplification, as posterior parts of the frontal cortex develop before the anterior regions, with the prefrontal cortex taking the longest to develop in primates (Fuster 2001; Burman et al. 2007). Our previous microarray analysis of human areal gene expression found twice as many gene probe sets to be relatively highly expressed anteriorly than those highly expressed posteriorly, but nevertheless the vast majority of genes showed no marked differences, including pan-neuronal markers such as synaptophysin, MAP2 and β-tubulin, and transcription factors important in development such as *PAX6, FEZF2,* and *SOX5*, which was confirmed by qPCR, ISH, and/or IHC (Ip et al. 2010; Ip et al. 2011). Therefore, there is no clear evidence for anterior regions maturing more quickly in terms of gene expression and differentiation. Nevertheless, there is differential gene expression across the early human neocortex (Ip et al. 2010) including arealization genes such as *EMX2, COUP-TFII,* and *FGFR3,* identified in rodent studies as important in establishing the protomap (O'Leary and Nakagawa 2002; Rakic 2009) and areally expressed genes that define the protomap, such as cell adhesion molecules that control cell migration and axon pathway finding (e.g., *CNTNAP2, PCDH17, ROBO1*) are more highly expressed anteriorly (Abrahams et al. 2008; Ip et al. 2010; Ip et al. 2011).

In addition to qPCR evidence, histological preparations showed that although there is evidence of migration from the GE to the cortex at earlier stages (7.5 PCW, Fig. 6*F*,*G*), we see no similar streams of cells at later stages (8–12 PCW). Furthermore, expression of ASCL1 to be high and prominent in the VZ, and high in the CP, which can be interpreted as evidence for intracortical generation of GABAergic interneurons, as ASCL1 expression is downregulated in migratory ganglionic eminence-derived interneurons before they enter the dorsal pallium (Letinic et al. 2002). Crucially, we co-localized ASCL1 expression to cells undergoing proliferation (Fig. 9). The expression of a number of other markers, including DLXs, CALB2, and GAD2 was also higher in the cortical wall than in the GE, a reverse of the gradient expected if inhibitory interneurons were predominantly migrating from subcortical to cortical structures. These markers were also strongly expressed in the SVZ, and we found dividing cells that were positive for DLX2. In a previous study, in organotypic cultures of forebrain at 10 PCW, 95% of newborn cells exhibit radial migration within the dorsal pallium and hardly any cells migrated tangentially across the subpallial/pallial border and never into the VZ (Letinic et al. 2002). Many GABAergic transcription factors (DLXs, NKx2.1, and LHX6) as well as GABA are detectable in cells of the VZ and SVZ, as well as outer layers, at this stage of development (Letinic et al. 2002; Rakic and Zecevic 2003; Jakovcevski et al. 2011) and the present study. Therefore, we propose that the anterior cortex is a genetically determined center for preferential generation of cortical interneurons. Differences in the capacity to generate GABAergic neurons from organotypic cultures of cortex (Letinic et al. 2002; Hansen et al. 2010) may depend on the anterior or posterior origin of the tissue.

We observed that the ventral pallium showed weaker expression of GABAergic markers compared with either the lateral/caudal GE or the dorsal pallium. A cloned neurogenic progenitor cell line produced from embryonic rat cortex generates interneurons in response to the ventral signaling molecule sonic hedgehog (Shh) however, this is suppressed by the dorsal signaling molecule Bmp2 (Li et al. 2008). Possibly, all mammalian cortex possesses the capacity to generate GABAergic interneurons when stimulated with ventralizing factors, as this can be achieved in vitro with rodent cortical tissue even if there is scant evidence of this occurring in vivo (Welagen and Anderson 2011). In human, our preliminary observations suggest that the ventral pallium may produce “dorsal” signaling molecules, as its pattern of gene expression resembles dorsal cortical hem (Sarma et al. 2013) which could locally inhibit interneuron generation. However, more dorsal and anterior regions come under the influence of the anterior neural ridge, which releases “ventral” signals (Monuki 2007). Holoprosencephaly is caused by disruption of genes contributing to the SHH-signaling pathway active in specifying rostroventral structures in the subpallium and results in selective loss of GABAergic cells characteristic of MGE derived interneurons in the neocortex (Fertuzinhos et al. 2009). If, in human, rostroventral gene expression domains also appear in the dorsal pallium then intracortical generation of GABAergic interneurons would also be disrupted by holoprosencephaly. It is known that genes implicated in this condition, for example, *FGFR1* and *FGFR2* (Fernandes and Hébert 2008) are expressed in the human fetal neocortex (Ip et al. 2010).

Primates display a complexity in layer I of the cortex not found in rodents (Meyer et al. 1998; Zecevic and Rakic 2001). In humans, around 11 PCW, the subpial granular layer (SGL) starts forming from cells observed to spread from the olfactory region to the nearby anterior/ventral cortex, and can be seen in sections in the present study at PCW12 (Fig. 8). By 13 PCW, the SGL covered the entire cortical surface of the forebrain, the SGL consists of small GABAergic cells and large Reelin-positive Cajal–Retzius cells; the GABAergic cells being able to undergo inward migration into the CP (Zecevic and Rakic 2001; Rakic and Zecevic 2003). During the time period studied in this experiment, the SGL could potentially provide a component of raised anterior GABAergic gene expression.

Also, from 10 PCW, increasing numbers of CALB2-positive neurons were observed in the IZ, SVZ, and VZ. Many show morphology distinctive of tangentially migrating neurons and may represent neurons migrating into the neocortex from ventral locations. In organotypic slice culture experiments, tangentially migrating cells were only observed in the SVZ/VZ in large numbers for the first time at 14 PCW, however, 70% of these expressed both DLX1/2 and ASCL1, and are thus likely to have been generated intracortically (Letinic et al. 2002). We propose that interneurons are generated intracortically predominantly in the frontal lobe and may migrate away from this site to populate other cortical areas. The cerebral cortex has expanded at a much faster rate than ventral telencephalic structures in the course of evolution of primates compared with rodents, and so it is logical that extra sources of interneurons might be required to populate the primate cortex. Furthermore, the frontal lobe in particular may show greater expansion in primates (Rakic 2009).

A proportion of human GABAergic forebrain neurons express the transcription factor COUP-TFII (Reinchisi et al. 2012). In the rodent, this transcription factor identifies interneurons of CGE origin and controls their migration via a caudal stream into the posterior cortex (Yozu et al. 2005; Kanatani et al. 2008; Miyoshi et al. 2010) and, in human, this gene is also predominantly expressed by interneuron precursors of the CGE compared with the MGE (Reinchisi et al. 2012). In the cerebral cortex, at mid-gestation, around a quarter of all GABAergic cells and three quarters of all CALB2-positive cells in the CP co-express COUP-TFII and a small proportion of dividing cells in the SVZ/VZ also express COUP-TFII. Intriguingly, there is a posterior/lateral to anterior gradient of COUP-TFII expressing cells at 15–22 PCW in the cerebral cortex, interpreted as caudal stream of migration of a population of interneurons generated in the CGE and probably the posterior and temporal cortical proliferative layers (Reinchisi et al. 2012). There also may be a lateral/posterior to anterior gradient of COUP-TFII expression at 8–12 PCW (http://brainspan.org/rnaseq/search/index.html) although at this stage, cells expressing the transcription factor seem to be largely confined to the presubplate and SP (Reinchisi et al. 2012) or the CGE. Therefore, the pronounced anterior to posterior gradient of CALB2 expression we observe at this time, which is augmented by the appearance of postmitotic CALB2 immunoreactive cells in the proliferative zones from 10 PCW, appears to derive from a different population than the COUP-TFII-expressing interneurons that appear at later stages.

### Conclusion

Our study provides substantial evidence for the capacity of human cortical progenitor cells to generate inhibitory interneurons. In addition to prior findings that this occurs before the formation of the CP, and also from midgestation, we now show that it may occur at the earliest stages of CP formation and to a greater extent towards the anterior pole of the cortex. This should provide interneurons to populate the lower layers of the CP which are forming at this time (Ip et al. 2011) which, in rodents, are predominantly derived from the MGE and characterized by expression of DLX transcription factors (Butt et al. 2005). At this stage, in human, expression of the *DLX*s actually appeared higher in the cortex than in the MGE by ISH (Fig. 3) and, in agreement with Letinic et al. (2002), we propose that tangential migration from the MGE to cortex starts at a later stage.

## Supplementary Material

Supplementary material can be found at: http://www.cercor.oxfordjournals.org/.

## Funding

N.A.-J. was supported by a scholarship awarded by the Iraqi Ministry of Higher Education and Scientific Research. The human embryonic and fetal material was provided by the Joint MRC/Wellcome Trust Human Developmental Biology Resource (grant # 099175/Z/12/Z; http://www.hdbr.org). Funding to pay the Open Access publication charges for this article was provided by the Wellcome Trust.

## Supplementary Material

Supplementary Data
