# Supplementary material for: The Early Fetal Development of Human Neocortical GABAergic Interneurons
Source: Cereb Cortex. 2013 Sep 18;25(3):631–45. doi: 10.1093/cercor/bht254 (PMC4318531; doi:10.1093/cercor/bht254)
Supplement: Supplementary Data [file supp_25_3_631__index.html]

The Early Fetal Development of Human Neocortical GABAergic Interneurons — The Early Fetal Development of Human Neocortical GABAergic Interneurons — Supplementary Data 

# The Early Fetal Development of Human Neocortical GABAergic Interneurons

## Supplementary Data

Supplementary Data

**Files in this Data Supplement:**

- Supplementary Data - Docx file
- Supplementary Figure 1 - tif file
