# Supplementary material for: The Early Fetal Development of Human Neocortical GABAergic Interneurons
Source: Cereb Cortex. 2013 Sep 18;25(3):631–45. doi: 10.1093/cercor/bht254 (PMC4318531; doi:10.1093/cercor/bht254)
Supplement: Supplementary Data [file supp_bht254_bht254supp.docx]

**Supplementary Figure 1. Comparison of anti-sense and sense ISH.**

**This figure demonstrates the specificity of the ISH probes used in this study (DLX1,2 and 5 and GAD1). Anti-sense probes gave clear patterns of expression, whereas there was no detection of the sense probes using identical staining protocols. Sections are from dorsal cortex at 10 PCW.**

**Scale bar = 200 μm**
