# Supplementary figures and images for: The Early Fetal Development of Human Neocortical GABAergic Interneurons
Source: Cereb Cortex. 2013 Sep 18;25(3):631–45. doi: 10.1093/cercor/bht254 (PMC4318531; doi:10.1093/cercor/bht254)

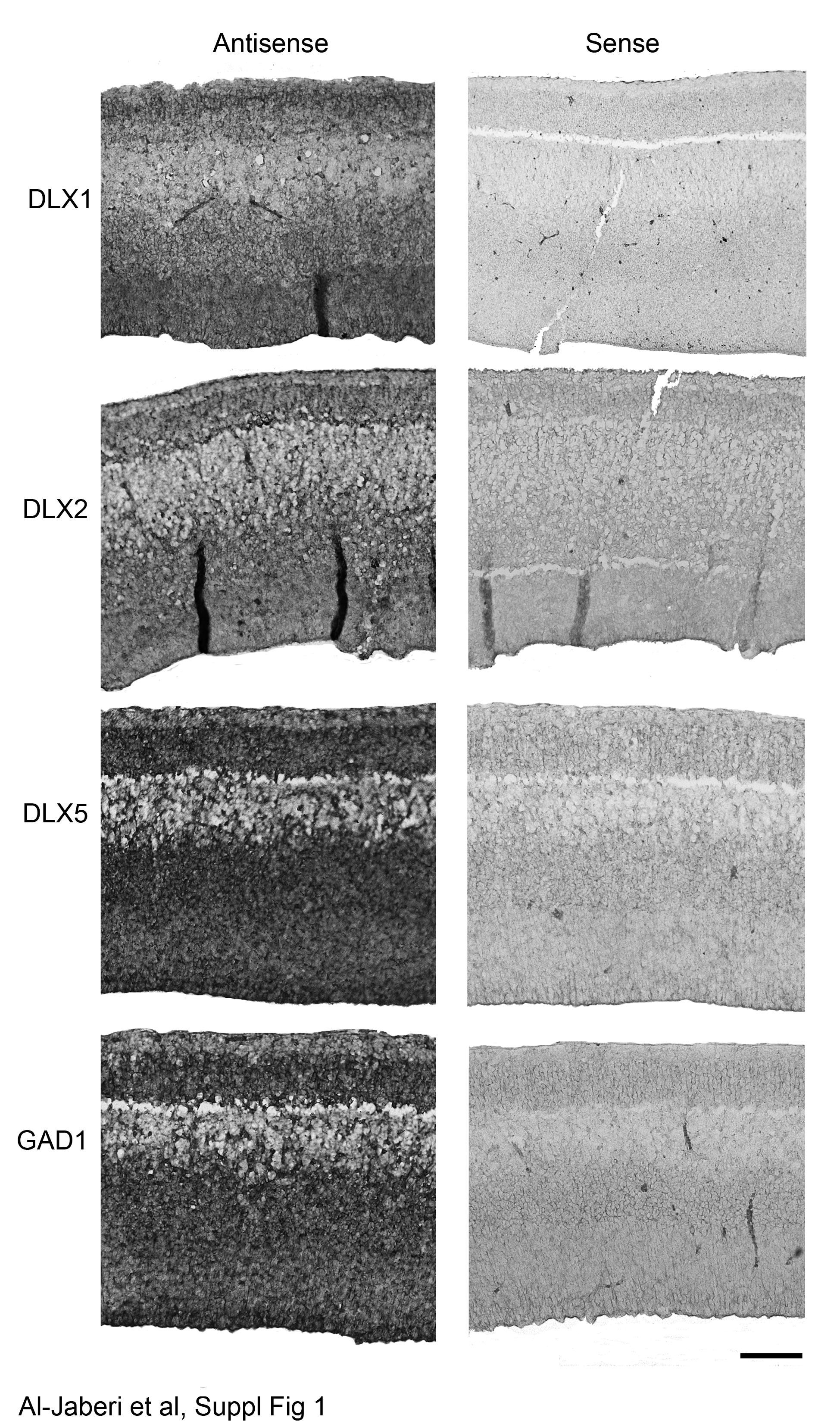

Supplement: Supplementary Data [file supp_bht254_bht254supp_fig1.tif]
